# Past body shaming experiences and food and alcohol disturbance in young adults: indirect effects via psychological distress

**DOI:** 10.1007/s40519-024-01687-z

**Published:** 2024-09-17

**Authors:** Daniele Di Tata, Dora Bianchi, Franca Rossi, Laura Maria Fatta, Stefania Sette, Fiorenzo Laghi

**Affiliations:** 1 Department of Developmental and Social Psychology, Sapienza University of Rome, via dei Marsi, 78, 00185 Rome, Italy; 2 Italian National Institute of Health, Rome, Italy

**Keywords:** Food and alcohol disturbance, Drunkorexia, Body image, Body shaming, Victimization, Psychological distress

## Abstract

**Purpose:**

This study investigated the associations between retrospective reports of body image victimization (i.e. body shaming) perpetrated by peers and by parents during childhood or adolescence, and food and alcohol disturbance (FAD) in young adulthood, considering the possible mediating role of psychological distress (i.e. subthreshold symptoms of anxiety and depression).

**Methods:**

The study involved 1624 young adults aged between 18 and 30 (69% women), who completed an online survey.

**Results:**

Our findings revealed that participants who reported more frequent body image victimization episodes during childhood and adolescence exhibited higher levels of psychological distress and, in turn, higher scores of FAD in young adulthood.

**Conclusions:**

This result represents a novel contribution to understanding the psychological correlates of FAD in youths. Limitations and implications are discussed.

*Level of evidence* Level V, descriptive study.

## Introduction

### Food and alcohol disturbance among young people

Food and Alcohol Disturbance (FAD), also named as drunkorexia, describes the use of any compensatory behavior to offset the calories consumed from alcohol and/or to amplify the intoxicating effects of alcohol [1]. Previous studies identified three dysfunctional behavioral patterns characterizing FAD: (1) self-imposed caloric restriction; (2) engaging in excessive physical activity; (3) adopting compensatory weight-control behaviors (e.g., vomiting or using laxatives) [2–5]. FAD is typically associated with body image concerns and the fear of gaining weight, leading individuals to modify their eating patterns to offset the caloric intake from drinking alcohol [6]. Moreover, this health-risk behavior has also been linked to a desire to intensify alcohol’s effects. [2]. In this regard, reducing caloric intake represents a strategy for quickly achieving the desired level of drunkenness. According to its underlying motives, FAD can occur before, during, or immediately after the drinking episodes [7]. For instance, individuals may engage in FAD before drinking to either reduce calorie intake or increase alcohol’s effects [8]. This health-risk behavior may persist during drinking for similar reasons. Afterward, negative feelings about potential weight gain may prompt compensatory behaviors [8]. Given the absence of a diagnosis for FAD in DSM-5-TR [9], researchers proposed specific operational criteria to guide clinical practice. Indeed, while it’s possible to have both eating disorders and alcohol abuse alongside FAD, the core of this behavior lies in its unique combinations of motivations, something not inherently found in other co-occurring conditions [1]. Additionally, although there is no official threshold for recognizing FAD, it has been proposed that compensatory behaviors should occur at least once a month to be considered indicative of FAD [10].

Previous research suggested that the incidence of FAD is high among young adults [11]. Specifically, among US college students the prevalence rates range between 10 and 55% [11]. Estimates in Italy confirmed this trend, indicating a prevalence of FAD of around 30% among young adults [12]. Furthermore, prior research reported mixed evidence about gender differences in the occurrence of this health-risk behavior [13]. Notably, previous studies on FAD prevalence have exclusively included participants reporting alcohol consumption, but have also demonstrated considerable heterogeneity in assessment methodologies, thus limiting the comparability of the results [11]. However, given that numerous studies reported a heightened risk of alcohol-related consequences among individuals exhibiting FAD [11], identifying the correlates of this behavior is essential for developing effective prevention programs.

### Body image victimization in eating disorders and alcohol use

FAD shares some psychological correlates with eating disorders, including psychological distress symptoms, and body image concerns [14–17]. Body image refers to an individual’s self-perception and attitude towards their body [18]. In this respect, recent studies show that FAD is linked to poor body esteem, body dissatisfaction, sociocultural attitudes toward appearance, drive for thinness, and internalization of body ideals [11, 19, 20]. Being victimized regarding physical appearance has been established indeed as a reliable predictor of disordered eating [21]. However, to our knowledge, no studies have yet investigated the association between body image victimization (BIV) and FAD.

BIV, also defined with the recent label of *body shaming* [22], refers to acts directed to harass or humiliate a person about their body shape [23, 24]. These experiences can occur across various contexts and could be perpetrated by different sources of influence, including peers and parents. Recent research highlights the mental health impacts of BIV, revealing its predictive role in the occurrence of maladaptive eating behaviors [25, 26]. To understand this relationship, the dual pathway model [27] proposes that sociocultural pressures regarding physical appearance, including BIV, can exacerbate body dissatisfaction by creating a gap between personal appearance and societal beauty standards. This dissatisfaction may drive individuals to attempt body changes engaging in disordered eating behaviors. Noteworthy, while most research has focused on BIV perpetrated by peers and its strong association with disordered eating [28], there is also evidence about parental BIV during childhood and adolescence [23, 29]. Previous studies found significant associations between parental BIV and various adolescent eating problems, including binge eating, emotional eating, and restrictive eating [29, 30]. However, to our knowledge, no study has yet investigated the association between parents’ and peers’ BIV and FAD in young adulthood.

Regarding alcohol consumption, literature indicates a positive correlation between alcohol misuse and body image concerns [31, 32]. Building upon this, prior research has found that body dissatisfaction in adolescence significantly predicts alcohol misuse in young adulthood [33, 34]. According to the self-medication theory [35], high levels of psychological distress due to body image concerns may prompt alcohol use to alleviate such negative feelings. Specifically, individuals excessively dissatisfied about their bodies may experience shame and anxiety in social situations. In such circumstances, drinking alcohol may serve as a maladaptive coping strategy to enhance confidence, easing social inhibition [36]. Moreover, individuals may also drink alcohol to alleviate emotional distress triggered by BIV experiences [37]. Nevertheless, to our knowledge, no study has yet investigated the possible association leading from peers’ and parents’ BIV to emotional distress and FAD in young adulthood.

### The role of psychological distress

Building on prior research that identified internalizing problems and low self-esteem as mediators in the relationship between victimization and eating disorders [38, 39], Lee and Vaillancourt [40] proposed a model outlining the pathway linking peer victimization to eating problems. The authors suggested that peer victimization negatively impacts children and adolescents’ psychological functioning, which, in turn, significantly predicts disordered eating. Similarly, previous evidence suggested that parental BIV may contribute to eating problems by influencing psychosocial functioning, negative self-evaluation, and symptoms of anxiety [29]. Moreover, there is evidence about the mediating role of psychological distress in the link between childhood victimization and alcohol use in adulthood [41]. Starting from this evidence, it is worth further investigating a possible indirect role of BIV experiences on FAD in young adults via the mediating effect of a general perceived psychological distress.

### Aims and hypotheses

The present study examined the pathways linking childhood and adolescent BIV, psychological distress, and FAD behaviors in young adulthood. Building on previous research linking BIV with eating disorders [28, 29] and alcohol use [37] we hypothesized significant associations between past BIV, perpetrated by peers and parents, and engagement in FAD in young adulthood. Moreover, following previous research on eating disorders [29, 38] and alcohol use [41], we also expected that childhood and adolescent BIV, by peers and parents, was positively associated with psychological distress in young adulthood which, in turn, was positively linked with FAD.

## Methods

### Participants and procedures

This study included 1624 young adults aged 18 to 30 (*M*_age_ = 23.82, *SD* = 2.87; 69% women). Inclusion criteria were set to include individuals aged 18–30 who were current alcohol consumers. Consequently, 152 participants who responded with 0 (*never*) to item 1 of the AUDIT questionnaire (i.e., “How often do you drink alcoholic beverages?”) and 31 participants older than 30 years were excluded from the study. Moreover, 17 participants who self-identified as nonbinary were excluded from the analyses because we could not make meaningful comparisons with the men and women groups. Initially, 1824 individuals agreed to participate providing informed consent. However, only 1624 young adults met the inclusion criteria and were included in the study. Regarding educational level, one person reported having a primary school diploma, 2% of the sample had completed middle school, 46% had finished high school, 47% had obtained a bachelor’s degree, and 5% had achieved a master’s degree. Concerning the participants’ occupation, 3% were unemployed, 24% held stable employment, 52% were engaged in academic studies, and 21% were concurrently employed and pursuing education. Data were gathered between December 2022 and November 2023 using a snowball sampling method, through an anonymous online survey publicly posted on the university website. This study was approved by the Ethics Committee of Sapienza University of Rome.

### Measures

#### Individual information

Participants reported their gender (coded as 0 = male, 1 = female, 2 = other), age, height, and weight. Additionally, information on education and occupation was collected.

#### Alcohol use

The Alcohol Use Disorders Identification Test (AUDIT-10) [42, 43] was used to evaluate participants’ patterns of alcohol use and alcohol-related problems. The instrument consists of 10 items (e.g., “How many drinks containing alcohol do you have on a typical day when you are drinking?”; *ω* = 0.89) addressing the quantity and frequency of alcohol consumption, and alcohol-related problems. Each item is rated on a response scale from 0 to 4.

#### Psychological distress

Perceived psychological distress was evaluated with the General Health Questionnaire-12 (GHQ-12) [44, 45]. The instrument consists of 12 items with a 4-point scale ranging from 0 (*less than usual*) to 3 (*no more than usual*), inquiring about subthreshold anxiety and depression symptoms experienced in the preceding two weeks (e.g., “In the past two weeks, have you felt unhappy or depressed?”; *ω* = 0.91).

#### Body image victimization

The Body Image Victimization Experiences Scale (BIVES) [22] was used to retrospectively assess childhood and adolescent experiences of BIV, perpetrated by peers and by parents. This measure consists of 12 items rated on a 5-point scale ranging from 1 (*never*) to 5 (*very frequently*) across two subscales: victimization by peers (6 items; e.g., “At school, I was left out/excluded because of my body shape”; *ω* = 0.96) and victimization by parents (6 items; e.g., “My mother/father said or did things that made me feel bad about my physical appearance”; *ω* = 0.97).

A blinded forward–backward translation procedure was conducted to adapt the original Portuguese version to the Italian context [46]. The final Italian version was included in the survey. A confirmatory factor analysis (CFA) was subsequently run on the collected data to verify the adequacy of the original two-factor model. The model exhibited an acceptable fit, confirming the presence of two correlated factors, Satorra-Bentler (S-B) *χ*^2^ (53) = 404.298, *p* < 0.001; CFI = 0.97; TLI = 0.96; SRMR = 0.03; RMSEA = 0.09; 90% CI [0.08, 0.10].

#### Food and alcohol disturbance

FAD was assessed using the Drunkorexia Motives and Behaviors Scale (DMBS) [47, 48]. This instrument comprises 23 items with a 5-point response scale ranging from 1 (*never*) to 5 (*always*), distributed across two subscales: Drunkorexia Motives (11 items; e.g., “On a day I planned to drink, I controlled my eating because my friends encourage me to restrict my calories”), which examines the reasons behind respondents’ engagement in FAD, and Drunkorexia Behaviors (12 items; e.g., “On a day I planned to drink, I controlled my eating by exercising before I drank”), evaluating various compensatory behaviors characterizing FAD. In order to examine the association between past BIV experiences and FAD behaviors, only the Drunkorexia Behaviors subscale was utilized. This subscale has shown good psychometric properties in previous international [47] and Italian studies [15, 16].

### Data analysis

Statistical analyses were conducted with R software [49], using the psych [50], lavaan [51], semTools [52], and sempower [53] packages. Preliminarily, descriptive statistics and bivariate correlations were computed. Subsequently, a CFA was conducted to inspect the psychometric properties of the latent factors of FAD, alcohol use, psychological distress, and BIV. The pattern of inter-item correlations indicated significant error covariances between items 15 and 17, and items 16 and 18 of the DMBS. Since these two pairs of items depict similar behavioral patterns of FAD, the residual covariances within each pair of items were included in the model.

Therefore, we estimated a structural equation model to examine the indirect pathways linking the two dimensions of childhood and adolescent BIV to FAD, via psychological distress. Participants’ gender, age, alcohol use, and BMI were included as covariates to control for their effects. Values of CFI and TLI above 0.90 and RMSEA and SRMR below 0.08 indicate an acceptable model fit [54]. Following Preacher and Selig [55], Montecarlo simulations with 20,000 iterations were computed to estimate the indirect effects and their 95% confidence intervals (CIs).

## Results

Descriptive statistics and bivariate correlations are reported in Table 1.

**Table 1:** Bivariate correlations and descriptive statistics among study variables

	*1*	*2*	*3*	*4*	*5*	*6*	*7*	*8*
1. Gender	–							
2. Age	− 0.11**	–						
3. BMI	− 0.17**	0.07**	–					
4. Alcohol use	− 0.11**	0.01	0.03	–				
5. BIV by peers	0.00	0.02	0.22**	0.06*	–			
6. BIV by parents	0.13**	− 0.07**	0.27**	0.09**	0.43**	–		
7. Psychological distress	0.14**	− 0.09**	0.04	0.08**	0.19**	0.23**	–	
8. FAD	0.06*	− 0.02	0.04	0.26**	0.21**	0.23**	0.14**	–
*M (SD)*	–	23.82 (2.87)	22.89 (3.87)	0.55 (0.42)	1.75 (1.00)	1.63 (1.03)	1.17 (0.55)	1.41 (0.78)
*Scale range*	–	18–30	14.84–46.87	0–4	1–5	1–5	0–3	1–5

Gender (0 = *Man*, 1 = *Woman*). *BMI* Body mass index, *BIV* Body image victimization, *FAD* Food and alcohol disturbance *M* Mean, *SD* Standard Devation

* *p* < 0.05, ** *p* < 0.01

**Fig. 1:**
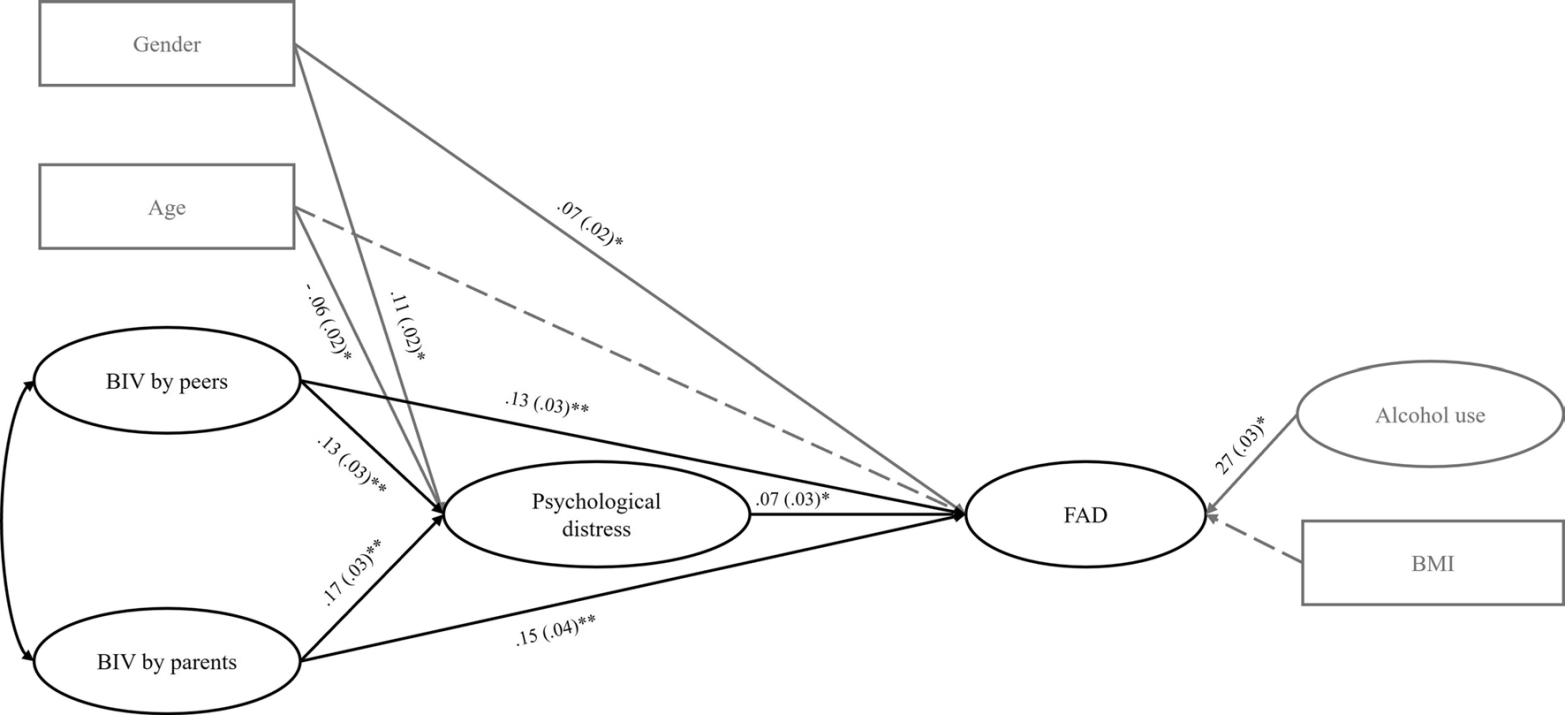
Links Between BIV, Psychological distress, and FAD behaviors. *Notes:* Solid lines represent significant paths, while dashed lines depict non-significant paths. Control paths are shown in grey. Gender (0 = Man, 1 = Woman).* BMI* body mass index,* BIV* body image victimization,* FAD* Food and alcohol disturbance. *p < .05, **p < .01

Results of the CFA indicated that the measurement model exhibited an acceptable fit to the data (S-B *χ*^2^(1100) = 4703.240, *p* < 0.001; CFI = 0.91; TLI = 0.90; SRMR = 0.04; RMSEA = 0.05; 90% CI [0.05, 0.05]). The structural equation model showed significant positive associations between the two dimensions of BIV, by peers and parents, and psychological distress. We also found significant positive associations between BIV by peers and parents and FAD. Additionally, psychological distress was positively and significantly correlated with FAD. The analysis of indirect effects revealed that psychological distress symptoms partially mediated the link between childhood and adolescent BIV by peers and FAD (*β* = 0.01, *SE* = 0.004, 95% CI [0.003, 0.019]). Similarly, psychological distress was found to partially mediate the association between BIV by parents and FAD (*β* = 0.01, *SE* = 0.004, 95% CI [0.004, 0.024]). Moreover, we found that women reported higher levels of psychological distress and FAD, compared to men. Results also showed a significant negative association between age and psychological distress and a positive and significant association between alcohol use and FAD. Finally, neither age (*β* = − 0.02, *p* = 0.42) nor BMI (*β* = − 0.03, *p* = 0.23) showed a significant association with engagement in FAD behaviors. Figure 1 displays all path standardized coefficients.

The structural model showed an acceptable fit to the data (S-B *χ*^2^ (1102) = 4904.176, *p* < 0.001; CFI = 0.91; TLI = 0.90; SRMR = 0.04; RMSEA = 0.05; 90% CI [0.05, 0.05]), and accounted for 16% of the variance in FAD and 9% in psychological distress. A post-hoc power analysis was performed to ensure the robustness of the model, revealing that power exceeded 99% at a significance level (*α*) of 0.05.

## Discussion

Given the recent surge in BIV among children and adolescents, it is crucial to investigate how these negative experiences affect the development of health-risk behaviors in young adulthood. Indeed, in recent years, BIV has become increasingly prevalent among young people, permeating various social contexts such as schools and social media [22]. For instance, a recent study estimated a prevalence of approximately 45% of BIV among a group of adolescents [24]. These experiences, common in adolescents with higher BMI, can lead to long-lasting negative effects on mental and physical health, increasing the risk of eating disorders and health-risk behaviors [56].

This study aimed to investigate the links between retrospective reports of childhood and adolescent BIV, perpetrated by peers and by parents, and FAD behaviors via the mediating role of psychological distress. Overall, the results confirmed our hypotheses, showing that young adults who reported more frequent BIV by peers and by parents during childhood and adolescence showed higher levels of psychological distress and FAD.

Additionally, psychological distress was found to partially mediate the associations between the two dimensions of BIV and FAD in young adulthood. The results extend previous findings about the positive impact of BIV on the development of problematic eating behaviors [25, 26] demonstrating that childhood and adolescent BIV is also linked with FAD in young adulthood. According to the dual pathway model [27], sociocultural pressures on physical appearance work in concert with various risk factors in affecting eating behaviors in young people. Experiencing BIV at home or school may prompt feelings of shame and inadequacy regarding body image, which, in turn, may result in poorer emotional well-being and greater use of weight-control behaviors [57]. Specifically, Chen et al. [58] found that BIV by parents and peers may result in increased body dissatisfaction and depressive symptoms, which, in turn, contribute to restrictive eating behaviors. In addition, our study sustains that psychological distress symptoms linked to BIV may push young individuals to adopt FAD, driven by the fear of gaining weight. Moreover, according to the self-medication theory [37], psychological distress associated with BIV may reinforce the use of alcohol for coping motivations. The desire to intensify alcohol effects for managing emotional distress is a key motivation for FAD [48]. Thus, our results seem to suggest that young adults may use FAD behaviors to amplify alcohol’s impact, in order to alleviate psychological distress feelings linked to past experiences of BIV [58]. It is noteworthy indeed that FAD typically occurs in social drinking contexts. Thus, these conducts may be moved by the need to gain self-confidence in social contexts and alleviate negative feelings about body inadequacy, which is specifically raised in comparison with others. These experiences might also be reinforced by an expected negative judgment of others on their own appearance, as a long-term consequence of child and adolescent BIV experiences. Future research should explore more in-depth these possible psychological mechanisms.

Thus, we align with previous studies on eating disorders [58] and alcohol use [41] providing evidence for the mediating role of psychological distress in the association between BIV and FAD. However, despite our findings indicating a comparable magnitude and similar pattern of association between peer- and parent-perpetrated BIV with psychological distress and FAD behaviors, further research is needed to determine if these experiences operate through different psychological routes, affecting the development of FAD and its motivations in different ways. Moreover, consistent with previous research [59, 60] we found higher levels of psychological distress symptoms and FAD behaviors among women compared to men. In line with recent evidence [61], we also found greater symptoms of depression and anxiety among younger individuals.

## Strengths and limits

Research on FAD has primarily focused on peer dynamics, overlooking the analysis of parental interactions. This study contributes to address this gap by offering novel evidence for the link between parental BIV and FAD and offering insights into the mediating role of psychological distress. Future research should examine other potential mediators such as low self-esteem and body dissatisfaction in the relation between BIV and FAD.

Nonetheless, the study displays some limitations. First, we employed a retrospective assessment of past BIV experiences. Future longitudinal research is essential to enhance our understanding of the causal pathway from past body shaming to young adults’ FAD. Second, our reliance on self-report measures introduced a potential for desirability bias in respondents’ answers. Future studies should implement a mixed-method approach to comprehensively evaluate FAD in young people. Third, the use of convenience sampling limits the generalizability of these findings. Fourth, we did not include control measures of eating psychopathology, nor did we examine the relationship between BIV, psychological distress, and the underlying motivations for FAD. Future research should incorporate a comprehensive assessment of eating psychopathology to control for the potential confounding effects of co-occurring eating disorders on FAD behaviors. Additionally, future investigations of the concurrent impact of childhood and adolescent BIV experiences and psychological distress on FAD motivations (i.e., offsetting alcohol-related caloric intake or enhancing intoxication) may significantly contribute to a deeper understanding of the developmental pathways leading to FAD behaviors. Finally, individuals with nonbinary gender identities were not included due to insufficient group size. To ensure inclusivity, future investigations on FAD should actively involve nonbinary individuals.

### What is already known on this subject?

Previous studies suggested that BIV represents a reliable predictor of disordered eating and alcohol use in young people. However, to our knowledge, no studies have investigated the link between BIV and FAD.

### What this study adds?

This study provides novel evidence about the association between BIV experiences and FAD, via the mediating role of psychological distress. Our findings suggest that managing the psychological distress stemming from BIV experiences could help to reduce FAD in young adults. In this regard, intervention programs should focus on training young people who suffer from BIV to use more adaptive strategies in responding to these situations. Specifically, it could be important to assist children and adolescents in developing critical thinking skills to discern the messages they receive about their body image. Promoting critical questioning of negative body image messages among young people could empower them to resist internalizing unrealistic aesthetic ideals and harmful evaluations of themselves, thereby reducing feelings of anxiety and depression associated with BIV experiences. Moreover, including mental health awareness campaigns and educational activities promoting body acceptance, respect, and body awareness in preventive programs, should help reduce the frequency of BIV among young people and mitigate their adverse effects on psychological distress and FAD behaviors.

## Data Availability

No datasets were generated or analysed during the current study.
